# Effects of Urban Park Quality, Environmental Perception, and Leisure Activity on Well-Being among the Older Population

**DOI:** 10.3390/ijerph182111402

**Published:** 2021-10-29

**Authors:** Yu-Ting Chu, Dongying Li, Po-Ju Chang

**Affiliations:** 1 Department of Horticulture and Landscape Architecture, National Taiwan University, Taipei 106, Taiwan; chu19950907@gmail.com; 2 Department of Landscape Architecture & Urban Planning, Texas A&M University, College Station, TX 77840, USA; dli@arch.tamu.edu

**Keywords:** depression, leisure activity, urban park

## Abstract

Previous studies have shown that natural environments and leisure activities can reduce depression and increase well-being. Urban parks are important for the psychological well-being of middle-aged and older adults. However, it remains unknown whether the relationship between environmental perceptions, leisure activity, and well-being is affected by the quality of park environments. This study uses a cross-level framework to examine the effects of urban park quality on middle-aged and older adults’ environmental perceptions, leisure activity, and well-being. The Neighborhood Green Space Tool was used to assess the environmental quality of 19 parks, and 380 individuals aged 55 years and older were interviewed in each park using an on-site questionnaire. The results reveal that the associations between environmental perception and well-being were moderated by the quality of park accessibility, amenities, and incivilities; the effect of environmental perception on depression was moderated by the quality of incivilities in parks; and the effect of frequency of leisure activities on depression was moderated by the quality of park accessibility.

## 1. Introduction

Under the global trend of urbanization and rapid population growth, nearly 80% of Taiwan’s population lives in urban areas [1]. With this growth of urbanization, human recreational needs are also increasing. Urban green space can satisfy recreational needs, but the current pace of urbanization has caused the loss or fragmentation of urban green space. With the rapid shrinkage of urban parks and green spaces, the quality of life and health conditions of urban residents are affected [2]. In addition, with the rapid aging of the population in recent years, the number of older people living in urban areas has been increasing, and the demographic structure of urban areas is very different from that of the past [3]. The social pressures of the urban environment, coupled with the physical and cognitive decline that comes with aging, as well as chronic illnesses, have led to higher levels of depression in older adults compared with other age groups [4]. Although the natural environment can bring many physical and psychological restorative benefits to humans, the sensory loss caused by aging not only affects the leisure activity patterns and psychobiological health of the older population, but it may also affect environmental perception and restorative experiences [5].

Parks are the most accessible natural spaces for middle-aged and older people in urban environments, and they can be used as places for leisure activities to promote well-being. However, the spatial layout, maintenance, and management vary from park to park, resulting in the differences in the quality of each park, which may have an impact on the perception of the environment and measures of well-being associated with participation in recreational activities.

### 1.1. Aging and Well-Being

With the advancement of technology and medical care, the average life expectancy has increased, leading to changes in the age structure at the population level. The older population aged 65 or older reached 14.5% in 2018, officially moving Taiwan into the “aged society” category [6]. The older population are faced with the stress caused by chronic diseases, loss of spouse or family, and life transitions, such as retiring and moving, which may lead to mental health crisis and depression [4]. In recent years, the suicide rate of the older population has gradually increased, and depression has become one of the main causes of suicidal tendencies [4].

In Taiwan, according to the definition of Article 2 of the Elderly Welfare Act, anyone who has reached the age of 65 or older is called an “elderly person. Article 53 of the Labor Standards Law also states that workers who have worked for more than 15 years and are 55 years old or older can retire; in 1993, the average retirement age of Taiwanese individuals dropped to 54.9 years old. Therefore, the “middle-aged and older adults,” as defined in the current study, is the population aged 55 years or older.

### 1.2. Leisure in Green Spaces

Previous studies have confirmed that engaging in leisure activities in a natural environment can modulate psychological well-being [7]. The higher the frequency of physical activity, the greater the decrease in depressive symptoms [8]. Natural environments attract people to outdoor activities and promote physical health by stimulating them to participate in outdoor activities [9,10]. Because middle-aged and older individuals tend to engage in regular leisure activities only near their homes, the activity patterns of middle-aged and older adults are closely related to their surrounding environmental patterns; therefore, physical environmental factors (e.g., walkways, seating facilities, lighting, etc.) are important predictors of physical activity in the aging population [11,12,13].

The concept of natural environmental quality can be applied to examine the mechanisms by which natural environments produce health benefits [2]. Urban parks are a type of natural environment, but there is not yet a unified set of assessment criteria for them because of the different types and scales of environments and the individual definitions of environmental quality in various studies [14]. Therefore, the Neighborhood Green Space Scale Assessment Tool (NGST, Neighborhood Green Space Tool) was developed to evaluate the quality of neighborhood green spaces. This tool contains six categories: accessibility, recreation, convenience, natural features, incivilities, and usability [14].

### 1.3. Environmental Perception and Well-Being

Regarding the benefits of the natural environment, subjective experience may be as important as the objective quality of the environment. A model was developed to illustrate the concept of environmental perception, here by using perceptual nature as an example [15]. Perceive nature refers to the discrepancy between the subjective cognitive nature and the objective real nature [15], which is generated by the processing of experience and knowledge after receiving information about the physical environment. Environmental perception is the subjective understanding and attitude of the human interpretation of environmental information, and there is a substantial discrepancy between the original objective and real environmental information. The Neighborhood Open Space (NOS) scale was developed to understand the relationship between the natural environment of a neighborhood and the quality of life and health of older adults [16].

A previous study also mentioned that the correlation between perceived green qualities (PGQ) and well-being was stronger than objective green values [17]. Residents’ perceptions of the environment may affect their subjective well-being more strongly and directly than their objective environmental qualities [18,19,20]. Related results can be found in a study examining the relationship between neighborhood naturalness and mental health that was conducted over a two-year period; here, subjects who showed an increase in perceptual naturalness over the two years also demonstrated reduced depressive symptoms [21].

## 2. Materials and Methods

### 2.1. Research Sites

**Figure 1:**
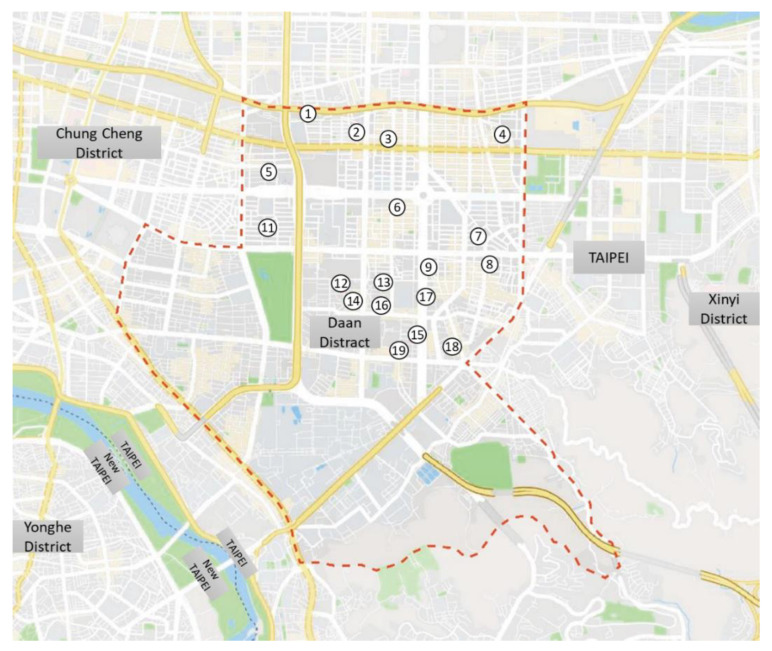
Selected neighborhood park locations (Number 1–19 referred to the parks in Table 1).

Because of the limited mobility associated with aging, middle-aged and older people tend to engage in outdoor recreational activities in parks near their homes. They also tend to visit parks frequently and at regular intervals to engage in recreational activities [22]. A neighborhood park is a green space with an area of less than one hectare, serves the residents of the neighborhood, and supports activities, such as resting and walking [23]. Therefore, the present study focuses on neighborhood parks as the study sites. Daan District in Taipei City has the highest level of aged individuals, with over 20% of the population aging. The total number of neighborhood parks in Daan District is 53. A three-step process was used to select research sites. First, we checked the demographic data of each ward in Daan District, analyzed the population aging index of each ward, and selected the wards with an aging index of more than 20% from these wards, totaling twenty-one neighborhood parks in this district were included. However, two of these parks were excluded because of construction inside the parks during the data collection. Therefore, the 19 neighborhood parks in Daan District, Taipei City, were used as the survey sites for this study (Table 1, Figure 1).

### 2.2. Participants

Of the middle-aged and older population in Taiwan, 83.5% are healthy or sub-healthy individuals (defined as a state of suboptimal health or experience between health and disease, with no significant physical disease) [24]. For this group, preventive care can be provided to maintain their psychosocial health. Therefore, individuals aged 55 years or older who were capable of independent mobility and self-care were used as the subjects. The study used an in situ questionnaire to invite a convenience sample of senior citizens in the park. Potential participants who were not local residents or under 55 years old were excluded. Included potential participants received a verbal brief about the purpose and procedure of the study, and after obtaining their consent, they were invited to assist in completing the questionnaire. Ethical review and approval were waived for this study due to the Announcement of the Department of Health, Executive Yuan Ministry of Health (Taiwan), Letter No. 1010265075: “The scope of human research cases that can be exempted from review by the Ethical Review Board when it is not based on minors, institutionalized persons, indigenous people, pregnant women, mentally and physically challenged persons, mentally ill persons, and others who have been determined or judged by the review board to be unduly coerced or unable to sit of their own free will.” The target population in this study is healthy park users aged 55 and above, which is not included in the above statement. Informed consent was obtained from all subjects involved in the study.

### 2.3. Procedure and Measurement

The data collection was completed in two parts: the first was an audit of the physical environment of the park by trained evaluators, and the second was the field survey. The NGST [24] was used to assess the quality of each of the 19 urban parks (Table 2), and the use of the tool was assessed by referencing its proprietary manual.

The NGST, a spatially appropriate assessment tool for neighborhood green spaces, contains six categories—accessibility (e.g., number of access, path quality), recreation (e.g., the quality and quantity of playground, field), amenities (e.g., quality of bench, trash can), natural features (e.g., quality of grass, tree, flower bed), uncivilized behavior (e.g., tracks of drinking, noise), and usability (e.g., walking, playing)—with 38 questions in total. There are 5 items of accessibility, 7 items of recreation, 4 items of amenities, 4 items of natural features, 8 items of uncivilized behavior, and 5 items of usability. Giblow et al. (2012) provided a detailed tool for coding and calculated each indicator. All items were scored from 1 = bad quality/hard to find/not at all) to 4 = good quality/everywhere/agree. All items of each category were summed to create the indicator. In addition, a series of formula of domain weights was provided: accessibility weight = (accessibility sum/11) × 100) × (18/100); recreation weight = (recreation sum/42) × 100) × (16/100); amenities weight = (amenities sum/16) × 100) × (22/100); natural feature weight = (natural feature sum/12) × 100) × (20/100); uncivilized behavior weight = (uncivilized behavior sum/14) × 100) × (24/100). The “Notice Indicators” category was incorporated into the “Amenities” category. Because the “Overall Impression” category includes the assessment of the aesthetics of open space and the safety of space, which involves a subjective evaluation in the measurement, the category was removed. The NGST is a more suitable tool for evaluating smaller green spaces (e.g., neighborhood parks) than other tools for assessing the quality of open space [24]. Overall, the NGST evaluates six types of park characteristics (i.e., access, recreational facilities, amenities, natural features, and incivilities), of which usage is used only as a distinction for park use type and is not included in the measurement of park quality. The details of the NGST evaluation can be found in Giblow et al. (2012).

An on-site questionnaire was used to investigate the effects of the perceptions of park environment and participation in recreational activities on the well-being of individuals at the individual level. It took about 15 min for participants to fulfill the questionnaire. The NOS scale [16] was used for the environmental perception scale. The Cronbach’s alpha value was 0.744 for a total of 13 questions. An example item is as follows: “There are many activities that can be generated here.” Two questions were asked about the frequency and duration of recent recreational activities in the park. Well-being was measured by life satisfaction and depression. Life satisfaction was measured by The Satisfaction with Life Scale (SWLS), which has five items [25]. The reliability of Cronbach’s alpha value was 0.850. An example item is “I am satisfied with my life.” The Geriatric Depression Scale (GDS-15) [26] was used for the depression scale. The reliability of the Cronbach’s alpha value was 0.840 for a total of 15 questions. An example item is “Have you recently cut back on your activities and hobbies?” The NOS, SWLS, and GDS-15 were measured by a Likert 5-point scale ranging from 1 (strongly disagree) to 5 (strongly agree). The items of each measure were averaged to create the indicator. Demographic and socioeconomic data (i.e., gender, age, education, marital status, and number of chronic diseases) were collected.

### 2.4. Theoretical Hypotheses and Analysis

**Figure 2:**
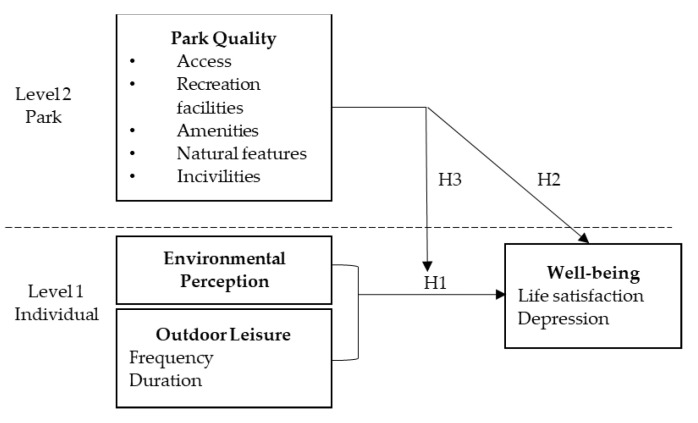
Theoretical framework and hypotheses.

The present study was conducted to investigate whether the well-being of middle-aged and older people’s perceptions of the park environment and their engagement in leisure activities differed depending on the quality of the park. Based on previous studies and the concept of multilevel modeling as a way to explore the relationship between the variables, a two-level modeling framework was used to account for the clustering of variables at the group (park) and individual levels to understand the relationship between park quality and individual well-being (H3), as well as the influence of environmental perception and leisure activity participation at the individual level on well-being (H1). The study further explored whether park characteristics moderated the relationships between perception, activity, and well-being (H2) (Figure 2).

Hierarchical linear modeling was used to examine the hypotheses. First, the null model was developed to examine whether there were significant differences in the mean values of participant data across groups and to determine whether it was appropriate to conduct subsequent hierarchical modeling. The two null models of life satisfaction and depression for the participants were constructed by placing the covariates “life satisfaction” and “depression” in the model and not placing any covariates at the participant level (individual level) and park level (group level), as below.

1. Level 1 Model: Life satisfaction _ij_/Depression _ij_ = *β*_0j_ + *r*_ij_;
2. Level 2 Model: *β*_0j_ = *γ*_00_ + *μ*_0j_; and
3. Mixed Model: Life satisfaction _ij_/Depression _ij_ = *γ*_00_ + *μ*_0j_ + *r*_ij_.

Next, the direct effect of the individual-level independent variables on the response could be predicted by substituting the life satisfaction/depression of the participants as the response, the environmental perception, activity frequency, and duration of the participants as the individual-level independent variables, and the individual background factors as the control variables. In the group level, the intercept and slope terms were substituted into the five environmental features of NGST to predict the direct and interactive effects of the group level on individual level, thus building a complete model of hierarchical linear regression, as shown below. The current study used the software HLM 6.08 to conduct the statistics and analysis of the data.

1. Level 1 Model:

Life satisfaction _ij_/depression _ij_ = *β*_0j_ + *β*_1j_ (environmental perception _ij_) + *β*_2j_ (leisure frequency _ij_) + *β*_3j_ (leisure duration _ij_) + *r*_ij_.

2. Level 2 Model:

*β*_0j_ = *γ*_00_ + *γ*_01_ (NGST feature _j_) + *μ*_0j_,

*β*_1j_ = *γ*_10_ + *γ*_11_ (NGST feature _j_) + *μ*_1j_,

*β*_2j_ = *γ*_20_ + *γ*_21_ (NGST feature _j_) + *μ*_2j_,

*β*_3j_ = *γ*_30_ + *γ*_31_ (NGST feature _j_) + *μ*_3j_.

3. Mixed Model:

Life satisfaction _ij_/depression _ij_

= *γ*_00_ + *γ*_01_ (NGST feature _j_)

+ *γ*_10_ (environmental perception _ij_) + *γ*_20_ (leisure frequency _ij_) + *γ*_30_ (leisure duration _ij_)

+ *γ*_11_ (NGST feature _j_ × environmental perception _ij_) + *γ*_21_ (NGST feature _j_ × leisure frequency _ij_) + *γ*_31_ (NGST feature _j_ × leisure duration _ij_) + *μ*_0j_ + *μ*_1j_ (environmental perception _ij_) + *μ*_2j_ (leisure frequency _ij_) + *μ*_3j_ (leisure duration _ij_) + *r*_ij_.

## 3. Results

### 3.1. Demographic Information

The present study included 19 parks in the Daan District of Taipei City and selected 20 active middle-aged and elderly people in each park to conduct questionnaire interviews. A total of 380 respondents were interviewed in the spring of 2019. Of the respondents, 94.5% had retired, and only 5.5% were in the labor force. In addition, 37.6% were men, and 62.4% were women, with women accounting for the majority of the participants who engaged in recreational activities in the park (Table 3). In the chronic disease section, 27.6% of middle-aged and elderly people did not have any chronic diseases, while 72.4% had at least one chronic disease and, at most, four diseases at the same time.

### 3.2. Quality of Neighborhood Green Space

According to the results of the NGST assessment (Table 4), each park’s total score on the six scales were calculated. Overall, the parks received similar scores on amenities and incivilities. Minh Rong Park and Siwei Park scored the highest on accessibility, while Ju An Park scored the lowest. The best amenity belonged to Changlong Park, which had an adequate number of seats and was one of the few neighborhood parks with trash receptacles, as well as good lighting at night. In the natural features category, Wenzhou Park had the highest score (9) among all the parks, with excellent maintenance of grass and shrubs and a pond in the park, which was relatively rare in the neighboring parks. In the noncivilized behavior assessment, the maintenance and management of the environment were all excellent, with eight parks achieving a full score (16) in this category. In terms of overall quality, Wenzhou Park was evaluated to have the best quality (71.12 points), and Ju An Park had the lowest quality (48.02 points).

### 3.3. Hierarchical Linear Modeling

#### 3.3.1. Null Model

In the null model with life satisfaction as the outcome (Table 5), the variance of the mean life satisfaction between parks was 0.027, and the chi-square test was used to determine that χ2(18) = 58.869, *p* < 0.001, indicating that the difference in mean life satisfaction between parks had reached a statistically significant level. In the null model with depression as the outcome, the difference in mean depression between parks was 0.128, χ2(18) = 56.270, and *p* < 0.001, indicating that the difference in mean depression between parks also reached a statistically significant level. In general, ICC1 values greater than 0.12 and ICC2 values greater than 0.60 [27,28] indicate significant differences between groups, while Cohen (1988) suggests that ICC1 values greater than 0.059 indicate that the variable is still moderately variable between groups. The ICC1 values of 0.102 and 0.096 for life satisfaction and depression for the participants were considered moderate differences. Therefore, it is appropriate to divide the life satisfaction and depression of the participants into two model levels.

#### 3.3.2. Hierarchical Regression

Table 6 shows the results of life satisfaction as the outcome. Five models were conducted to examine the different interactions. Among the direct effects of the individual-level independent variables on life satisfaction, environmental perception and leisure duration had positive and significant effects on life satisfaction for the middle-aged and elderly, but leisure frequency had no significant effect on life satisfaction. Among the direct effects of group-level variables on life satisfaction, only access had a positive and significant effect on life satisfaction (γ01 = 0.045, t = 2.509, *p* < 0.05), while the other four environmental features of NGST had no significant effect. Among the cross-level interaction effects, the variables access (γ11 = −0.107, t = −2.597, *p* < 0.05) and amenities (γ11 = −0.148, t = −3.546, *p* < 0.01) showed negative effects on the relationship between environmental perception and life satisfactory. However, uncivilized behavior (γ11 = 0.085, t = 4.733, *p* < 0.001) had a significantly positive effect. Recreational facilities and natural features had no significant moderating effect on the environmental perception–life satisfaction relationships.

Table 7 shows the results of the analysis, with depression as the outcome. Among the direct effects of the individual-level independent variables on depression, environmental perception and leisure frequency had negative and significant effects on depression; however, leisure duration had no significant effect. Among the direct effects of the group-level variables on depression, only access had a significantly negative effect on depression (γ01 = −0.051, t = −4.607, *p* < 0.001), while the other four environmental features of NGST had no significant effect on depression among the participants. Among the cross-level interactions, the interaction between access and leisure frequency of the participants (γ21 = −0.018, t = −2.328, *p* < 0.05) and between uncivilized behavior and environmental perception of the participants were significantly negative (γ11 = −0.079, t = −3.265, *p* < 0.01), while the direct effects of recreational facilities, amenities, and natural features on the individual level were not significantly moderated.

## 4. Discussion

This study uses a cross-level framework to examine the effects of urban park quality on middle-aged and older adults’ environmental perceptions, leisure activity, and well-being. The results reveal that the associations between environmental perception and well-being were moderated by the quality of park accessibility, amenities, and incivilities; the effect of environmental perception on depression was moderated by the quality of incivilities in parks; and the effect of frequency of leisure activities on depression was moderated by the quality of park accessibility.

### 4.1. Environmental Perception of Life Satisfaction among Senior Citizens and Accessibility in Parks

In previous studies, access to parks has shown a positive relationship in the life satisfaction of users [29], with higher perceptions of the environment also predicting greater life satisfaction [21]. Both of these relationships were confirmed in the present study, indicating that higher park accessibility and more positive attitudes toward park perceptions among middle-aged and older adults were associated with higher life satisfaction. However, the interaction between park accessibility and environmental perceptions in this age group had a negative moderating effect on life satisfaction, suggesting that although there was a positive influence of environmental perceptions on life satisfaction, the effects decreased as park accessibility increased. These results differ from those of previous studies. For example, a few studies on park accessibility in Western countries have focused on the distance between park users’ homes and the park, and the life satisfaction of park users who only need to walk to the park is higher than that of users who must drive to the park [29,30,31]. These discrepancies may be attributable to the differences in park size and proximity: the neighborhood parks in Taipei City are smaller in size than many Western neighborhood parks, with 53 neighborhood parks located within the 11.36 km^2^ in the Daan District and with the nearest neighborhood park being within walking distance of the middle-aged and older adults. They might just take a walk to the nearby parks after having dinner and stay only half an hour. Therefore, they can be satisfied with the small size of the parks, even if there are fewer amenities, because they are near their homes.

The NGST assesses the quality of “accessibility” features in parks only in terms of the quantity and quality of existing environmental features; however, because it is an objective assessment method, it cannot take into account the influence of the subjective preferences of middle-aged and elderly people. Therefore, this difference may be the reason for the negative moderating effect of park accessibility. In addition to the number of entrances and exits, the number of paths into the park, and the number and quality of trails in the park, the physical attributes of the environment, such as the type of entrances and exits and the width, length, color, and material of trails, may affect the subjective attitude of middle-aged and older people toward the environment. Because of the degeneration of middle-aged and older people’s joints based on the aging process, their preference for the physical attributes of the environment was safety, so they pay more attention to the details that may cause falls during park activities [32].

### 4.2. Environmental Perception of Life Satisfaction among Senior Citizens and Amenities in Parks

The results show that the interaction between the quality of park amenities and the environmental perceptions of the participants had a negative moderating effect on life satisfaction, representing the positive effect of environmental perceptions on life satisfaction, which decreases as the quality of park amenities increases; however, the quality of park amenities does not predict the life satisfaction. That is, the quality of park amenities does not predict life satisfaction. Few studies have examined the benefits of amenities in urban parks from the perspective of well-being [11,12,13]. While the NGST evaluated the quantity and quality of park amenities in terms of “seating,” “trash cans,” “trash cans (bags) for dog excrement,” and “lighting,” the major amenities in neighborhood parks in Taipei were “seating” and “lighting.” The evaluation method was based on the size of the park space to determine the adequacy of each amenity and the quality of the amenity. As reflected in the results, the quality of the park amenities did not have a significant effect on well-being [33]. Most users in the neighborhood park are middle-aged and older people, after the provision of amenities is able to satisfy the needs of them, even if the quantity and quality of the facilities are increased, it might not affect their life satisfaction.

Urban parks are natural spaces that provide residents with leisure and recreation opportunities and that can improve psychological health and quality of life [34]. As urban living standards improve and as residents’ demand for equipment and facilities inside parks grows, parks’ functions of supporting human activities will grow [35]. Therefore, as the number of amenities increases, the proportion of green space within neighborhood parks will tend to decrease, which may be the reason for the positive effect of environmental perception on the life satisfaction of the elderly regarding the negative quality of amenities.

### 4.3. Contributions, Limitations, and Future Suggestions

Because of the aging society in Taiwan, the users of neighborhood parks are mostly middle-aged and older people. Therefore, in the future, whether in the maintenance and management of neighborhood parks or in the improvement and design of spaces, more consideration can be given to the needs of middle-aged and older people and the psychological benefits that neighborhood parks can offer. The results show that the quality of parks contributed to the well-being of middle-aged and older adults, but the quantity and quality of park features, such as accessibility, amenities, and uncivilized behavior, are more influential in increasing the life satisfaction and reducing depression. Therefore, these three characteristics can be considered in depth for future improvements to the park environment. The results show that the NGST is an effective tool for assessing the quality of urban neighborhood parks and that its measures of park quality can predict the well-being of middle-aged and elderly people. In particular, middle-aged and older adults are the main users of neighborhood parks; hence, this tool can be used by community groups, government agencies, and private organizations that are particularly concerned about the park environment to assess the environmental quality of local neighborhood parks and to serve as a reference for making the community environment a healthy aging space. Three instructions for planning administrations who design and manage neighborhood parks were provided based on the results as follows.

(1) Accessibility: The design of neighborhood parks can be improved by providing clear and safe crosswalks or sidewalks and vehicle control signs on the pathways to the parks, in order to consider the safety of middle-aged people on their way to the parks. The entrances and exits of the park should take into account the scale of the park and maximize the degree of openness, while the installation should be transparent and obvious, and reduce the drop and obstruction at the entrances and exits of the park, and set up barrier-free facilities so that the middle-aged and elderly can enter and exit the park easily.

(2) Amenities: In addition to considering the needs of the middle-aged and older adults, the number of park amenities should be appropriately adjusted to the size of the park, and a certain degree of green space should be retained in order to maintain the buffering function of urban parks as natural spaces and to bring psychological benefits to residents.

(3) Uncivilized behavior: The quality of uncivilized behavior in parks mainly focuses on the maintenance and management of the park environment. Although it is necessary to emphasize the moral concept of park users and their centripetal force for space maintenance in this quality maintenance, it is recommended that the park space be improved by focusing on the permeability of the vision in order to reduce the incidence of uncivilized behavior in the park.

Regarding the limitations, the results cannot be extrapolated to all middle-aged and older adults in parks. To control for the psychological effects of major physical illnesses, middle-aged and older people who self-rated as unhealthy were not included as respondents. Therefore, future studies can further investigate the well-being of the middle-aged and older adults with a disease burden and the characteristics of the park environment that this group values more. In addition, the quality of the urban parks in the present study was evaluated by the five items of the NGST. The quality of the environmental features of the parks was only evaluated in certain aspects, and the detailed attributes commonly found within urban parks can be explored in depth in subsequent studies. Finally, the current study was a cross-sectional study with an on-site self-reported survey. Those who were less depressed or more satisfied with life might be more likely to visit and like the parks. Therefore, subsequent studies can sample more neighborhood parks and find subjects in other administrative areas of Taipei to diversify the data and make the multilevel model more comprehensive.

## 5. Conclusions

The current study has adopted a cross-level framework to investigate the effect of the group level (e.g., urban park quality) on individual level (e.g., middle-aged and older people’s environmental perceptions and the effect of leisure activity participation on well-being). The NGST was used to assess the physical environmental quality of 19 parks in Daan District, Taipei City. Here, 380 middle-aged people aged 55 years or older were interviewed in each park using a field questionnaire.

The results show that at the individual level, environmental perception and duration of leisure activities had a positive effect on life satisfaction. Environmental perception and frequency of leisure activities could reduce depression. However, in the cross-level interaction analysis, the effect of environmental perception and leisure activity participation on well-being did not change depending on the quality of urban parks. Therefore, we further subdivided urban park quality into the five characteristics of NGST, finding that the effect of environmental perception on life satisfaction was moderated by the quality of park accessibility, amenities, and noncivilized behavior; the effect of environmental perception on depression was moderated by the quality of noncivilized behavior; and the effect of leisure activity frequency on depression was moderated by the quality of park accessibility. Therefore, the subjective well-being of middle-aged and older people is affected by specific features of parks. Therefore, in future park design or maintenance management, focusing on these features will help improve the well-being of middle-aged and older people.

## Figures and Tables

**Table 1:** List of neighborhood parks used in this study.

Park	Area (m^2^)	Park	Area (m^2^)
Changlong	3807	2. Heian	1614
3. Rukong	1416	4. Dunan	2820
5. Minsheng	2896	6. Longjin No.1	1451
7. Ningxin	2080	8. Longjin No.2	2056
9. Minghui	1397	10. Siwei	3097
11. Daan	2913	12. Andong	1630
13. Yanji	2284	14. Longtou	1234
15. Gunhyeon	869	16. Wenzhou	1287
17. Jeonan	1575	18. Yan Ai	1390
19. Ju An	2089		

**Table 2:** Photos of research sites.

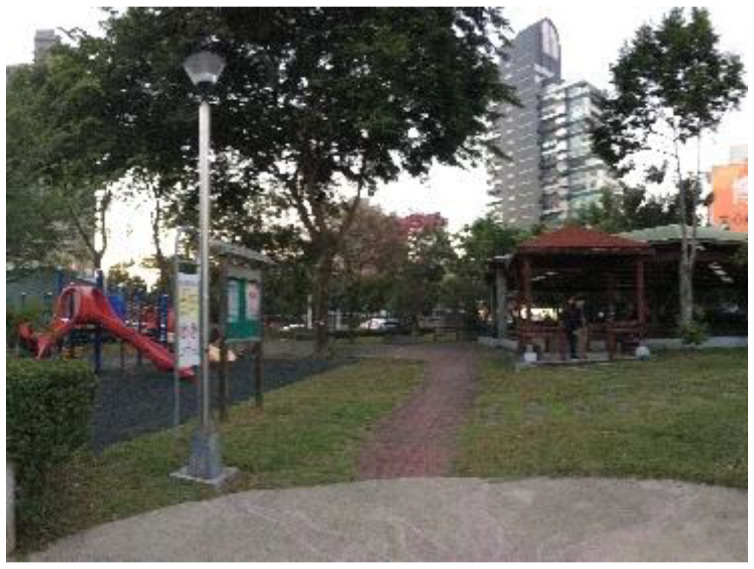	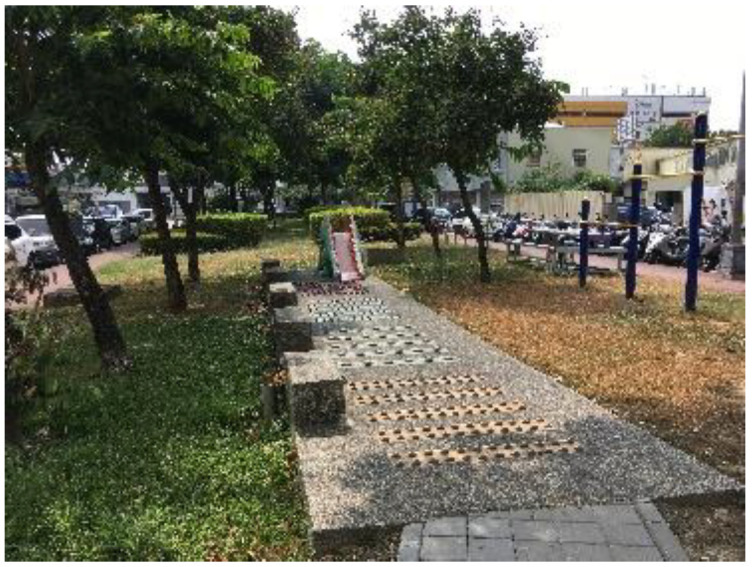	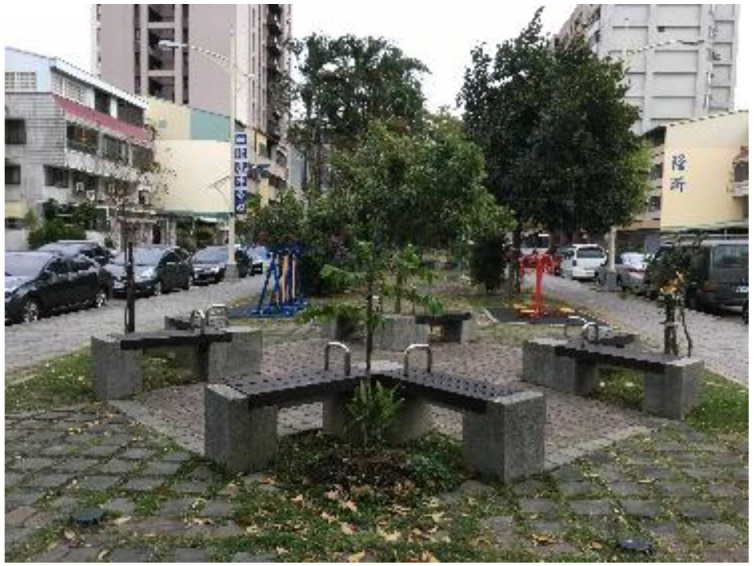
Changlong Park	Rukong Park	Minsheng Park

**Table 3:** Descriptive statistics of individual-level subjects’ background.

Variable	Item	Frequency	%
Gender	Female	237	62.4
	Male	143	37.6
Age	55–64	68	17.9
	65–74	203	53.4
	75–84	88	23.1
	85 and above	21	5.6
Education	Elementary	46	12.1
	Junior high	58	15.3
	Senior high	142	37.4
	College	125	32.9
	Master and above	9	2.4
Marital status	Single	10	2.6
	Married	299	78.7
	Divorced	38	10.0
	Widowed	33	8.7
Number of chronic diseases	0	105	27.6
	1	82	21.6
	2	106	27.9
	3	69	18.2
	4	18	4.7

**Table 4:** Summary of the neighborhood park quality assessment results.

Park	Participants	Neighborhood Green Space Tool Evaluation Results					
	N	Access	Recreation	Amentites	Nature	Incivilities	Total
Changlong	20	8.00	4.00	8.00	5.00	11.00	52.81
Rukong	20	6.00	15.00	6.00	6.00	15.00	59.50
Minsheng	20	10.00	18.00	6.00	6.00	16.00	68.90
Ningxin	20	6.00	10.00	6.00	5.00	16.00	57.64
Minghui	20	5.00	14.00	5.00	5.00	16.00	56.15
Daan	20	8.00	17.00	6.00	7.00	15.00	65.20
Yanji	20	8.00	16.00	4.00	6.00	16.00	62.11
Gunhyeon	20	5.00	14.00	6.00	5.00	14.00	54.10
Jeonan	20	8.00	17.00	6.00	6.00	16.00	65.25
Ju Aan	20	3.00	11.00	6.00	4.00	14.00	48.02
Heian	20	8.00	14.00	6.00	3.00	14.00	55.67
Dunan	20	9.00	17.00	6.00	6.00	15.00	65.17
Longjin No.1	20	7.00	19.00	6.00	6.00	16.00	64.37
Longjin No.2	20	8.00	19.00	5.00	4.00	15.00	59.58
Siwei	20	10.00	20.00	5.00	5.00	15.00	64.91
Andong	20	6.00	16.00	6.00	6.00	15.00	59.88
Longtou	20	5.00	16.00	5.00	6.00	16.00	58.58
Wenzhou	20	9.00	15.00	6.00	9.00	16.00	71.12
Yan Ai	20	6.00	13.00	6.00	6.00	14.00	57.02

**Table 5:** Summary of the null model for life satisfaction and depression.

Life Satisfaction			
Fixed Effect	Coefficient	*t*	D.F
Intercept			
Level 2 Mean	3.988	87.186 ***	18
Random effect	Variation	*χ* ^2^	D.F
Level 2 Variation in mean value among park	0.028	58.869 ***	18
Level 1 Variation in mean value in park	0.243		
Depression			
Fixed effect	coefficient	*t*	D.F
Intercept			
Level 2 Mean	2.070	58.023 ***	18
Random effect	Variation	*χ* ^2^	D.F
Level 2 Variation in mean value among park	0.128	56.270 ***	18
Level 1 Variation in mean value in park	0.393		

Note: D.F. = degree of freedom; *** *p* < 0.001.

**Table 6:** Moderating effect of NGST for each feature of the examined model (life satisfaction as an outcome).

Variable	Model 1	Model 2	Model 3	Model 4	Model 5
	Coefficient (*t*)	Coefficient (*t*)	Coefficient (*t*)	Coefficient (*t*)	Coefficient (*t*)
Intercept	3.718 (53.934 ***)	3.710 (51.670 ***)	3.713 (51.255 ***)	3.705 (50.230 ***)	3.707 (51.805 ***)
Direct effect at individual level					
Environmental perception	0.402 (4.994 ***)	0.390 (5.441 ***)	0.411 (5.810 ***)	0.373 (4.341 ***)	0.417 (6.234 ***)
Duration	0.088 (3.081 **)	0.089 (2.948 **)	0.086 (3.103 **)	0.081 (2.950 **)	0.082 (2.753 *)
Direct effect at group level					
NGST feature	0.045 (2.509 *)	0.009 (1.000)	0.007 (0.167)	−0.005 (−0.166)	0.017 (0.992)
Interaction between levels					
Access * environmental perception	−0.107 (−2.597 *)				
Access * leisure duration	0.005 (0.297)				
Recreation * environmental perception		0.014 (0.800)			
Recreation * leisure duration		−0.004 (−0.653)			
Amentites * environmental perception			−0.148 (−3.546 **)		
Amentites * leisure duration			0.039 (1.989)		
Nature * environmental perception				0.059 (1.094)	
Nature * leisure duration				0.028 (1.783)	
Incivities * environmental perception					0.085 (4.733 ***)
Incivilities * leisure duration					0.015 (0.623)
−*2LL*	490.359	503.870	492.809	495.608	493.950

Note: −2LL = dispersion coefficient, * *p* < 0.05, ** *p* < 0.01, *** *p* < 0.001.

**Table 7:** Moderating effect of NGST for each feature of the examined model (depression as an outcome).

Variable	Model 1	Model 2	Model 3	Model 4	Model 5
	Coefficient (*t*)	Coefficient (*t*)	Coefficient (*t*)	Coefficient (*t*)	Coefficient (*t*)
Intercept	2.221 (41.777 ***)	2.225 (43.549 ***)	2.220 (43.546 ***)	2.222 (40.339 ***)	2.223 (42.637 ***)
Direct effect at individual level					
Environmental perception	−0.364 (−4.516 ***)	−0.377 (−5.307 ***)	−0.387 (−5.387 ***)	−0.347 (−4.286 ***)	−0.391 (−5.814 ***)
Frequency	−0.116 (−5.580 ***)	−0.105 (−4.260 ***)	−0.110 (−4.774 ***)	−0.111 (−4.939 ***)	−0.104 (−4.087 ***)
Direct effect at group level					
NGST feature	−0.051 (−4.607 ***)	−0.020 (−2.102)	0.050 (1.679)	−0.005 (−0.309)	−0.016 (−1.155)
Interaction between levels					
Access * environmental perception	0.018 (0.531)				
Access * leisure duration	−0.018 (−2.328 *)				
Recreation * environmental perception		−0.006 (−0.350)			
Recreation * leisure duration,*γ_31_*		0.002 (0.419)			
Amentites * environmental perception			0.085 (2.097)		
Amentites *leisure duration			−0.040 (−1.987)		
Nature * environmental perception				−0.070 (−1.528)	
Nature *leisure duration				−0.018 (−1.432)	
Incivities * environmental perception					−0.079 (−3.265 **)
Incivilities * leisure duration					0.012 (0.844)
−*2LL*	286.814	299.320	291.888	299.567	295.739

Note: −2LL = dispersion coefficient, * *p* < 0.05, ** *p* < 0.01, *** *p* < 0.001.
